# Association among Premenstrual Syndrome, Dietary Patterns, and Adherence to Mediterranean Diet

**DOI:** 10.3390/nu14122460

**Published:** 2022-06-14

**Authors:** Yu-Jin Kwon, Da-In Sung, Ji-Won Lee

**Affiliations:** 1 Department of Family Medicine, Yongin Severance Hospital, Yonsei University College of Medicine, Yongin 16995, Korea; digda3@yuhs.ac; 2 Department of Medicine, Yonsei University College of Medicine, Seoul 03722, Korea; roselinasung@yonsei.ac.kr; 3 Department of Family Medicine, Severance Hospital, Yonsei University College of Medicine, Seoul 03722, Korea

**Keywords:** premenstrual syndrome, Mediterranean diet, dietary habit

## Abstract

Premenstrual syndrome (PMS) adversely affects the physiological and psychological health and quality of life of women. Mediterranean diet (MD) could be helpful for managing and preventing PMS, but evidence on the association between dietary patterns and PMS in Asian women is limited. This study aimed to investigate the association of dietary patterns and adherence to MD with PMS in Korean women. This cross-sectional study recruited 262 women aged 20–49 years via an online survey. PMS was diagnosed using the American College of Obstetricians and Gynecologists diagnostic criteria. MD adherence was assessed using the Korean version of the Mediterranean Diet Adherence Screener. Mediterranean Diet Score (MDS) was classified into tertiles (T) (T1: 0–3, T2: 4–5, and T3: ≥6). Dietary pattern was assessed with the Food Frequency Questionnaire. Multiple logistic regression analyses were conducted to evaluate the association between dietary pattern scores and PMS prevalence. The proportion of PMS was significantly lower in MDS tertile (T) 3 than in T1 (55.4% in T3 vs. 74.4% in T1, *p* = 0.045). After adjusting for confounders, participants in the highest tertile of the bread/snack pattern had a higher risk of PMS (odds ratio [95% CI]: 2.59 [1.32–5.06]), while traditional dietary pattern and meat/alcohol pattern were not associated with PMS. In conclusion, we found that low adherence to MD and higher bread/snack dietary pattern were associated with increased risk of PMS, respectively.

## 1. Introduction

Premenstrual syndrome (PMS), which occurs during the luteal phase of the menstrual cycle and resolves spontaneously after the onset of menstruation, is a common psychological and somatic disorder among women of reproductive age [1]. This syndrome consists of psychiatric or physical symptoms that significantly impair the normal daily functioning of women at any stage in her reproductive life, including work, relationships, and personal activities, and negatively impact quality of life [1,2,3]. Although the exact etiology of PMS remains unknown, the symptoms may be related to hormonal variations [4]. Genetic backgrounds, psychosocial factors, and lifestyle factors such as aerobic exercise and dietary pattern are known to associated with prevalence of PMS [1,5,6,7]. For example, excessive consumption of sweet-tasting food, fast food, deep-fried meals, coffee, and alcohol are significantly associated with the development of PMS [8,9]. In contrast, adequate intake of vegetables and fruits could alleviate PMS symptoms [8,9].

The Mediterranean diet (MD) is characterized by high consumption of vegetables, fruits, whole grains, legumes, nuts, and olive oil. Importantly, it has consistently been validated for its beneficial influence on health [10,11]. Thus, the MD could have beneficial effects on management and prevention of PMS [12]. However, there has been a limited amount of research investigating the association between dietary patterns and PMS in Asian women. Therefore, we aimed to investigate this association, particularly between PMS and MD adherence.

## 2. Materials and Methods

### 2.1. Study Design and Population

This cross-sectional study was conducted among Korean women of childbearing age who participated in an online survey in September 2021. The survey was conducted using survey panels from dataSpring (https://ko.d8aspring.com/contact). A total of 262 women aged 20–49 years voluntarily participated in the online survey. The exclusion criteria were as follows: (1) age <20 years or >49 years; (2) known history of thyroid disease; (3) current medical history of oral contraceptives or hormone replacement therapy; and (4) unwillingness to provide informed consent.

### 2.2. Data Collection

Data were collected during the online survey using a self-administered questionnaire. The participants were first assigned to a pre-screening procedure. The pre-screening questionnaire included anthropometric variables (height and weight), demographic characteristics, underlying medical conditions (hypertension, diabetes mellitus, and dyslipidemia), and menstrual history. Body mass index (BMI) was calculated as weight (kg) divided by height (m) squared. Menstrual history included age of menarche (years), menstrual length (days), duration of flow (days), menstrual regularity (regular/irregular), amount of flow (little/moderate/heavy), and dysmenorrhea (present/none).

Demographic information included age (years), education status (under-graduate or graduate), marital status (single or married), and personal lifestyle habits (smoking, alcohol drinking, and physical activity). Smoking and alcohol drinking habits were classified as never/former/current smoker and never/current drinker, respectively. Physical activity was assessed using the Godin Leisure-Time Exercise Questionnaire [13]. Participants were asked to recall the number of strenuous, moderate, or mild physical activity exceeding 15 min in duration, considering a 7-day period (a week). The intensity of each physical activity was described as “heart beats rapidly (e.g., running or vigorous swimming)”, not exhausting (e.g., fast walking, easy bicycling, and folk dancing)”, and “minimal effort (e.g., yoga or easy walking)”. Total weekly leisure activity score was calculated by multiplying the number of 15-min episodes by the weights of 9, 5, and 3, and summing those values into an overall score. Based on the total score, participants were categorized into three groups: active (≥24 units), moderately active (14–23 units), and insufficiently active/sedentary (<14 units).

The subjects who qualified in the pre-screening procedure proceeded to the main survey. The main survey questionnaire included: (1) American College of Obstetricians and Gynecologists (ACOG) diagnostic criteria for PMS [14,15]; (2) Korean version of the Mediterranean Diet Adherence Screener (K-MEDAS) [16]; and (3) Food Frequency Questionnaire (FFQ) [17].

### 2.3. Diagnosis of Premenstrual Syndrome

PMS was confirmed following the ACOG diagnostic criteria proposed in 2000 [14], as follows. PMS was diagnosed if the patient reported at least one affective symptom (i.e., depression, angry outbursts, irritability, anxiety, confusion, or social withdrawal) and at least one somatic symptom (i.e., breast tenderness, abdominal bloating, headache, or swelling of extremities) during the 5 days before menses in each of the three prior menstrual cycles. In addition, these symptoms should have been relieved within 4 days from the onset of menses, without recurrence until at least cycle day 13. The ACOG PMS questionnaires translated into Korean by the National Institute of Food and Drug Safety Evaluation [15] were used. Based on the ACOG diagnostic criteria, participants with or without PMS were classified into the PMS and non-PMS groups.

### 2.4. Assessment of Mediterranean Diet Adherence

Adherence to the MD was assessed using a 14-item questionnaire developed by Ji-won Lee et al. which was a modification of the validated Mediterranean Diet Adherence Screener [16]. The K-MEDAS questionnaire is a validated tool for assessing adherence to the Mediterranean diet in the Korean population. One point is given for using perilla oil or olive oil as the principal source of fat for cooking (question; Q1) and for preferring white meat over red meat (Q13). One point is also given in the following cases: for Q2, ≥3 teaspoons of perilla oil or olive oil per day; for Q3, ≥2 servings of vegetables per/day; for Q4, ≥1 pieces of fruit per day; for Q5, <1 serving of red meat or sausages per day; for Q6, <1 serving of butter, margarine, or cream per day; for Q7, <1 serving of sugar-sweetened beverages per day; for Q8, ≥7 servings of wine per week; for Q9, ≥3 servings of beans or tofu per week; for Q10, ≥3 servings of fish or seafood per week; for Q11, <3 times of sweets, bread (except whole wheat bread), cakes, and cookies per week; for Q12, ≥3 times of nuts per week; and for Q14, ≥3 times of whole grain per week. Failure to meet each parameter is given a 0 score. The resulting modified MD scores ranged from 0 to 14, with higher score indicating higher adherence to the MD.

### 2.5. Assessment of Nutritional Intake and Dietary Patterns

The nutritional survey was conducted using the semi-quantitative FFQ, which has been validated for 112 food items [17]. Participants were required to report the frequency of usual food items consumed in the preceding year on a daily, weekly, or monthly basis, considering the average amount of intake for each food item. Based on the nutrient composition similarity, food items were categorized into 21 food groups: rice, mixed grain rice, noodles/dumplings, breads/rice cakes, soup/stew, soybeans, eggs, red meat, white meat, fish/seafood, vegetables, fermented foods, kimchi, seaweed, potatoes, milk/dairy products, fruits, beverages, snacks, nuts, and alcoholic beverages. Total energy and nutrient intake values were derived from this FFQ. Detailed information regarding the specific food items is available on the KNHANES website [18].

### 2.6. Statistical Analysis

Normally distributed continuous variables were presented as the mean ± standard deviation or median (25th, 75th), while categorical variables were presented as the number (percentage, %). Between-group comparisons were performed using an independent two-sample t-test or Mann–Whitney U test for continuous variables and using chi-square test for categorical variables. For continuous variables, analysis of variance was performed to compare the differences among tertile groups. Dietary patterns were generated by factor analysis using the principal component method. Twenty-one food items were entered into the factor analysis. Eigenvalues > 1.0 were used to determine the number of factors to retain by scree plot. To increase interpretability, factors were rotated by orthogonal transformation (varimax).

Foods with factor-loading > 0.5 were considered to contribute to the dietary patterns. Finally, 15 food items were used in the factor analysis. Three major dietary patterns were extracted, which explained 49.5% of the total variance. The Keiser–Meyer–Olkin (KMO) value was 0.826. Factor scores for each dietary pattern were categorized into tertile. Multiple logistic regression analyses were conducted to evaluate the association between each dietary pattern score and prevalence of PMS. Model 1 was an unadjusted model. Model 2 was adjusted for age and BMI. Model 3 was adjusted for all factors in model 1 with the addition of smoking status, alcohol drinking status, and physical activity. All statistical analyses were performed using Statistical Package for the Social Sciences version 23.0 (IBM Corp., Armonk, NY, USA). All tests were two sided, and *p* values < 0.05 were considered statistically significant.

## 3. Results

### 3.1. Baseline Participant Characteristics

In total, 171 (65.3%) and 91 (34.7%) participants did and did not have PMS, respectively. The baseline participant characteristics by group are shown in Table 1. There were significant between-group differences in age, BMI, education, marital status, physical activity, and underlying conditions. The participants with PMS were likely to be current smokers and current drinkers (*p* = 0.046 and *p* < 0.001, respectively). Table 2 shows the menstrual characteristics by group. Although there were no differences in age of menarche, menstrual length, duration of flow, menstrual regularity, and amount of flow (little/moderate/heavy), the prevalence of dysmenorrhea was significantly higher in the PMS group (95.3% vs. 80.2%, *p* < 0.001).

### 3.2. Nutritional Status and Dietary Patterns

Table 3 presents the nutritional intake status according to the presence of PMS. Compared with the non-PMS group, the PMS group had a significant higher intake of total calorie, carbohydrate, fiber, vitamin A, vitamin K, and Ca. However, after adjusting for the total calorie intake, no significant difference in nutritional status between two groups was observed. Weekly consumption frequency of food groups in accordance with the presence of PMS is shown in Table 4. A comparison of weekly consumption frequency showed a significant difference with respect to the food groups of white meat, snacks, and alcoholic beverages. Weekly consumption frequency of white meat was significantly higher in the PMS group than in the non-PMS group (0.32 ± 0.46 times/wk vs. 0.23 ± 0.17 times/wk, *p* = 0.024). In addition, the PMS group showed significantly higher weekly consumption frequency of snacks (1.52 ± 2.07 times/wk vs. 1.05 ± 1.49 times/wk, *p* = 0.033) and alcoholic beverages (0.44 ± 0.86 times/wk vs. 0.25 ± 0.45 times/wk, *p* = 0.022).

**Figure 1:**
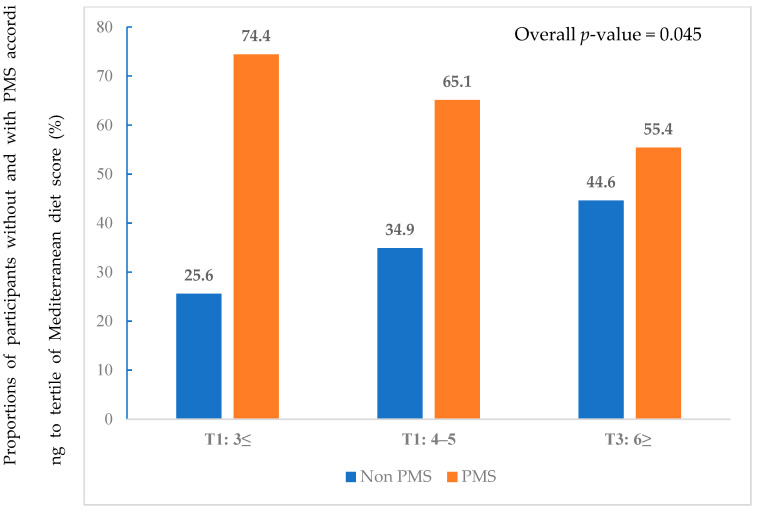
Proportions of participants without and with PMS according to Mediterranean diet score tertiles.

**Figure 2:**
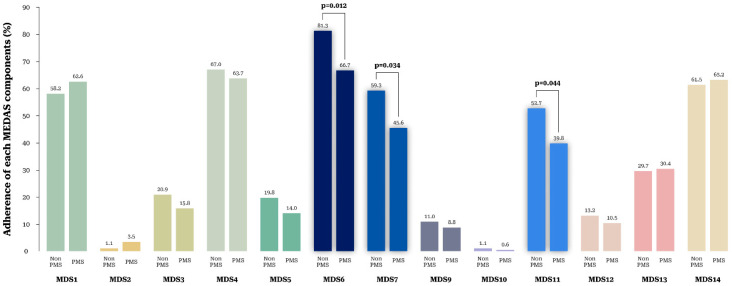
Proportion of participants without and with PMS according to adherence to each of the 14 components of the Mediterranean diet score.

Figure 1 presents the proportions corresponding to each tertile. MDS was classified into tertiles (T1: 0–3, T2: 4–5, and T3: ≥6). The proportions of PMS was significantly lower in T3 than in T1 (55.4% vs. 74.4%, *p* = 0.045), while the proportion of non-PMS was significantly higher in T3 than in T1 (44.6% vs. 25.6%, *p* = 0.045). Figure 2 shows the proportion of PMS according to adherence to each of the 14 MDS components. The non-PMS group showed a higher adherence rate to MD component 6 (<1 serving of butter, margarine, or cream per day) (81.3% vs. 66.7%, *p* = 0.012), component 7 (<1 serving of sugar-sweetened beverages per day) (59.3% vs. 45.6%, *p* = 0.034), and component 11 (<3 times of sweets, bread (except whole wheat bread), cakes, and cookies per week) (52.7% vs. 39.8%, *p* = 0.044). There were no significant differences in adherence to other components. Adherence to MDS component 8 (≥7 servings of wine per week) was zero for all participants.

### 3.3. Association between Dietary Patterns and PMS

We identified three major dietary patterns using factor analysis: (i) traditional diet pattern, which was high in fish/seafood, vegetables, kimchi, seaweed, potatoes and fruits; (ii) meat/alcohol pattern, which was high in rice, noodles/dumplings, red meat, and alcoholic beverages; and (iii) bread/snack pattern, which was high in breads/rice cakes and snacks. The identified dietary patterns explained 49.5% of dietary food intakes in the study population. The factor loadings associated with 15 food groups for the major dietary patterns are presented in Table S1.

Table 5 presented the odds ratio (OR) and 95% confidence intervals (CI) for PMS across tertiles (T) of the three major dietary patterns. The bread/snack pattern was associated with prevalence of PMS in model 1 (T3 vs. T1, OR, 95% CI 2.63 (1.39–5.00), *p*-value = 0.003). After adjusting for age, body mass index, smoking status, alcohol drinking status, and physical activity, this significant association was remained (T3 vs. T1, OR, 95% CI, 2.59 (1.32–5.06), *p*-value = 0.006). Traditional diet pattern was not associated with prevalence of PMS in model 1 (T3 vs. T1, OR, 95% CI 0.91, (0.49–1.68), *p*-value = 0.905). Meat/alcohol pattern was not associated with prevalence of PMS in model 1 (T3 vs. T1, OR, 95% CI, 1.82 (0.97–3.40), *p*-value = 0.060). After adjusting for same confounders, similar trends were shown (traditional diet pattern; T3 vs. T1, OR, 95% CI, 1.13 (0.56–2.30), *p*-value = 0.735 and meat/alcohol pattern; T3 vs. T1, OR, 95% CI 1.60 (0.83–2.09) *p*-value = 0.160).

## 4. Discussion

Research on the association between dietary patterns and PMS in Asian women is scarce. This cross-sectional study found that participants with PMS have lower adherence to MD. In addition, we identified three dietary patterns among Korean women: bread/snack, traditional diet, and meat/alcohol diet. Importantly, bread/snack dietary pattern was adversely associated with PMS, whereas traditional dietary pattern and meat and alcohol diet pattern were not associated with PMS.

A recent systemic review and meta-analysis conducted in India and Turkey reported that PMS was prevalent in 43–52.2% of women of reproductive age [19,20]. A Spanish cohort study also reported a PMS prevalence of 73.7% [21] and a meta-analysis in Iranian women of reproductive age reported a prevalence rate of 70.8% [95% CI: 63.8–77.7] [22]. This difference in prevalence among the studies might be due to the difference in the study population, and cultural contexts, as well as differences in study methodology such as sample size, design, and methods of PMS measurement. In our study, majority of participants had PMS (65.3% of 262 participants). Although it cannot be excluded that ACOG diagnostic criteria overestimated the prevalence of PMS [23], the rate was consistent with the results of previous studies that reported a high prevalence of PMS in Korea [24,25].

Dietary factors are influential but modifiable parameters that can be included in the management of PMS, and several studies have found a significant association between dietary habit and PMS [5,26]. A study conducted in the United Arab Emirates reported a high prevalence of PMS among university students with high calorie/fat/sugar/salt food consumption [26]. Western dietary pattern, which is characterized by high consumption of fast foods, soft drink, and desserts, is significantly associated with a higher likelihood of PMS, whereas healthy and traditional dietary pattern is associated with a lower risk [5,9]. The MD is rich in high-complex carbohydrate and monounsaturated fatty acids, rather than simple sweets and saturated and trans-fatty acids [10,11]. Many studies have shown that the MD has beneficial effects in preventing chronic diseases, such as metabolic syndrome, type 2 diabetes, inflammatory disease, cardiovascular disease, and cancer [27]. In a recent Spanish study, low adherence to the MD was associated with longer menstrual cycles [28]. Women who ate less than two pieces of fruit per day had a higher risk of menstrual pain (OR: 2.984; 95% CI = 1.390–6.406; *p* < 0.05) [28]. The amount of menstrual bleeding was lower in women who consumed olive oil daily, while it was more severe in those who consumed ham weekly.

However, only few studies have found a significant association between MD habit and PMS, especially in Asians. Our findings are consistent with those of previous studies. We found a significantly higher MD score in the non-PMS group than in the PMS group. The proportions of participants consuming butter, margarine, or cream less than one serving/day; sugar-sweetened beverage less than one serving; and sweets, bread, cakes, and cookies less than three times per week were significantly higher in the non-PMS group than PMS group. Further, highest bread/snack pattern was associated with a 2.59 times higher risk of PMS. Although the exact etiology of PMS still remains unknown, several possible mechanisms could support our findings. First, high consumption of bread and snacks with excess sodium [29] may decrease serum magnesium (Mg) levels [30,31].

Reduced Mg levels have been reported in women with PMS [32], and a randomized clinical trial confirmed that Mg supplementation alleviated PMS symptoms related to mood [32]. The traditional Korean diet pattern is characterized by high consumption of vegetables, legumes, whole grains, fish and fermented foods (kimchi, soy sauce, and soybean paste), and these components are similar to those of MD [33]. However, traditional fermented foods of Korean diet also contain high sodium [34]. Therefore, in the current study, traditional diet pattern might not be associated with prevalence of PMS. Second, individuals with PMS are prone to have depressive mood and stronger cravings for refined carbohydrate and fat foods [35,36]. Third, a western dietary pattern is closely related to chronic low-grade inflammation, and several studies proved significant positive associations between serum levels of inflammatory markers (e.g., high-sensitivity C-reactive protein, interleukin-12, and interferon-γ) and menstrual symptom severity in women with PMS [37,38]. In addition, the common psychiatric features of PMS have been associated with chronic inflammation [38]. Fourth, a previous study demonstrated that oxidative stress results from an oxidant/antioxidant imbalance is closely associated with various symptoms of PMS [39]. MD, a representative antioxidant diet, has abundant polyphenols and monounsaturated and polyunsaturated fatty acids. Therefore, MD could help to reduce PMS symptoms.

Our study has some limitations. First, the causal relationship between dietary patterns and PMS could not be investigated owing to the nature of cross-sectional study. Second, nutritional status and dietary pattern were assessed using the FFQ, which has disadvantages of lacking accuracy for absolute nutrient values, especially micronutrients and possibility of over and underreporting of consumption of certain foods [40]. In addition, dietary patterns do not represent all possible patterns and people may fit into more than one pattern. Third, we only included the participants who participated in the online survey panel. Therefore, our results have limited generalizability to the overall population of Korean women, other races, and other countries. Fourth, during the survey, we allowed subjects to freely report their diseases in addition to hypertension, dyslipidemia, diabetes, and cardiovascular disease. Participants who have mental illness are likely to understate their situation and report falsely. Therefore, we could not fully evaluate the individuals’ psychological status. Finally, the effect of unmeasured factors such as physiological condition (e.g., menopause status) and comorbidities cannot be completely ruled out. Despite these limitations, to our best knowledge, this study is the first to investigate the association between dietary pattern and PMS and examine adherence to MD as it relates to the prevalence of PMS among Korean women. Further investigations into the biological mechanisms by which dietary factors influence the pathogenesis of PMS are required.

## 5. Conclusions

This is the first study investigating the association between dietary pattern and PMS among Korean women. Our result suggested that bread/ snack pattern is associated with higher prevalence of PMS in Korean women. We also suggest that low adherence to MD is associated with PMS. Our findings suggest that adherence to MD and avoiding bread/snack consumption could be helpful for managing and preventing PMS. Further investigations into the biological mechanisms of dietary factors’ action on the pathogenesis of PMS are required.

## Figures and Tables

**Table 1:** Baseline participant characteristics by PMS group.

Characteristics	Non-PMS Group	PMS Group	*p*-Value
N	91 (34.7%)	171 (65.3%)	
Age (years)	33.0 (26.0–37.0)	31.0 (26.0–37.0)	0.213
BMI (kg/m^2^)	21.1 (19.6–23.1)	20.5 (19.1–22.9)	0.460
Education, N (%)			
Undergraduate	26 (28.6%)	46 (26.9%)	0.773
Graduate	65 (71.4%)	125 (73.1%)	
Marital status, N (%)			
Single	63 (69.2%)	123 (71.9%)	0.647
Married	28 (30.8%)	48 (28.1%)	
Smoking status, N (%)			
Never smoker	78 (85.7%)	126 (73.7%)	0.046
Former smoker	8 (8.8%)	19 (11.1%)	
Current smoker	5 (5.5%)	26 (15.2%)	
Alcohol drinking status, N (%)			
Never drinker	31 (34.1%)	23 (13.5%)	<0.001
Current drinker	60 (65.9%)	148 (86.5%)	
Physical activity, N (%)			
Active	22 (24.2%)	55 (32.2%)	0.389
Moderately active	28 (30.8%)	45 (26.3%)	
Insufficiently active/sedentary	41 (45.1%)	71 (41.5%)	
Underlying conditions, N (%)			
Hypertension	3 (3.3%)	1 (0.6%)	0.122
Diabetes mellitus	0 (0.0%)	3 (1.8%)	0.554

PMS, premenstrual syndrome.

**Table 2:** Menstrual characteristics of the study participants by PMS group.

Characteristics	Non-PMS Group	PMS Group	*p*-Value
Age of Menarche (years)	14.0 (13.0–15.0)	13.0 (12.0–13.0)	0.227
Menstrual Length (days)	28.0 (28.0–30.0)	29.0 (28.0–30.0)	0.275
Duration of flow (days)	5.0 (5.0–7.0)	6.0 (5.0–6.0)	0.771
Menstrual regularity, N (%)			
Regular	61 (67.0%)	124 (72.5%)	0.354
Irregular	30 (33.0%)	47 (27.5%)	
Amount of flow, N (%)			
Mild	10 (11.0%)	18 (10.5%)	0.993
Moderate	72 (79.1%)	136 (79.5%)	
Heavy	9 (9.9%)	17 (9.9%)	
Dysmenorrhea, N (%)			
Present	73 (80.2%)	163 (95.3%)	<0.001
None	18 (19.3%)	8 (4.7%)	

PMS, premenstrual syndrome.

**Table 3:** Nutritional status by PMS group.

Variables	Non-PMS Group	PMS Group	*p*-Value
N	91	171	
Total calorie	1667.6 ± 931.2	1950.6 ± 1121.5	0.041
Carbohydrate (g/day)	255.7 ± 128.3	295.7 ± 168.8	0.049
Protein (g/day)	45.9 ± 4.8	43.5 ± 3.3	0.112
Fat (g/day)	45.5 ± 34.3	55.1 ± 39.2	0.050
Fiber (g/day)	10.7 ± 1.1	13.3 ± 1.0	0.036
Saturated fatty acids (%)	1.93 ± 0.2	1.90 ± 2.0	0.584
Polyunsaturated fatty acids (%)	5.36 ± 2.90	5.35 ± 1.95	0.961
Monounsaturated fatty acids	5.34 ± 1.90	5.50 ± 2.20	0.539
Omega-3 fatty acids	0.30 ± 0.31	0.29 ± 0.20	0.692
Omega-6 fatty acids	0.01 ± 0.02	0.01 ± 0.01	0.783
Omega-6/Omega-3	0.08 ± 0.19	0.06 ± 0.11	0.268
Vitamin A	298.6 ± 236.4	386.2 ± 304.2	0.011
Vitamin C	93.4 ± 83.5	115.2 ± 121.1	0.088
Vitamin D	3.2 ± 5.0	4.2 ± 11.5	0.473
Vitamin E	13.4 ± 10.7	15.9 ± 11.5	0.098
Vitamin K	116.1 ± 121.0	166.2 ± 177.9	0.008
Riboflavin	1.3 ± 0.9	1.5 ± 1.0	0.250
Niacin	9.8 ± 7.3	11.5 ± 7.2	0.075
Vitamin B6	1.7 ± 3.8	1.5 ± 1.0	0.660
Ca	375.2 ± 280.0	460.3 ± 307.9	0.029
Na	2327.4 ± 2287.3	2783.7 ± 2068.3	0.103
K	2216.4 ± 1547.6	2584.9 ± 1665.6	0.082
Zinc	8.6 ± 7.7	9.7 ± 5.8	0.222

PMS, premenstrual syndrome.

**Table 4:** Weekly consumption frequency (times/week) of food groups by PMS group.

Variables	Non-PMS Group	PMS Group	*p*-Value
N	91	171	
Rice	2.31 ± 1.67	2.43 ± 1.73	0.598
Mixed grain rice	5.19 ± 5.88	5.04 ± 5.67	0.84
Noodles/dumplings	0.50 ± 0.49	0.58 ± 0.58	0.27
Breads/rice cakes	0.42 ± 0.48	0.51 ± 0.49	0.166
Soup/stew	0.36 ± 0.56	0.40 ± 0.41	0.492
Soybeans	0.67 ± 0.98	0.75 ± 1.08	0.56
Eggs	1.91 ± 1.89	1.90 ± 1.74	0.953
Red meat	0.48 ± 0.66	0.47 ± 0.44	0.833
White meat	0.23 ± 0.17	0.32 ± 0.46	0.024
Fish/seafood	0.38 ± 0.68	0.37 ± 0.61	0.957
Vegetables	0.59 ± 0.70	0.64 ± 0.74	0.623
Fermented foods	0.83 ± 1.65	0.96 ± 1.37	0.53
Kimchi	2.64 ± 3.00	2.92 ± 3.22	0.492
Seaweed	0.69 ± 1.37	0.66 ± 1.16	0.876
Potatoes	0.34 ± 0.64	0.35 ± 0.66	0.986
Milk/dairy products	1.37 ± 1.55	1.72 ± 1.97	0.11
Fruits	0.51 ± 0.53	0.57 ± 0.60	0.401
Beverages	1.80 ± 1.65	2.03 ± 1.72	0.289
Snacks	1.05 ± 1.49	1.52 ± 2.07	0.033
Nuts	0.19 ± 0.51	0.34 ± 1.35	0.171
Alcoholic beverages	0.25 ± 0.45	0.44 ± 0.86	0.022

PMS, premenstrual syndrome.

**Table 5:** Odds ratio and 95% confidence intervals for PMS according to the major dietary patterns.

	Traditional Diet Pattern		Meat and Alcohol Pattern		Bread and Snack Pattern	
	OR (95% CI)	*p*-Value	OR (95% CI)	*p*-Value	OR (95% CI)	*p*-Value
Model 1						
T1	1.00 (Ref)		1.00 (Ref)		1.00 (Ref)	
T2	1.07	0.831	1.75	0.076	1.91	0.039
	(0.57–2.00)		(0.94–3.26)		(1.03–3.53)	
T3	0.91	0.905	1.82	0.060	2.63	0.003
	(0.49–1.68)		(0.97–3.40)		(1.39–5.00)	
Model 2						
T1	1.00 (Ref)		1.00 (Ref)		1.00 (Ref)	
T2	1.11	0.751	1.80	0.068	1.87	0.048
	(0.59–2.09)		(0.96–3.36)		(1.01–3.48)	
T3	1.03	0.927	1.86	0.053	2.61	0.003
	(0.54–1.98)		(0.99–3.49)		(1.37–4.97)	
Model 3						
T1	1.00 (Ref)		1.00 (Ref)		1.00 (Ref)	
T2	1.17	0.635	1.53	0.205	1.88	0.056
	(0.61–2.29)		(0.79–2.95)		(0.98–3.58)	
T3	1.13	0.735	1.60	0.160	2.59	0.006
	(0.56–2.30)		(0.83–2.09)		(1.32–5.06)	

Ref, reference; PMS, premenstrual syndrome; OR, odds ratio; 95% CI, 95% confidence interval. Model 1: unadjusted model. Model 2: Adjusted for age and body mass index. Model 3: Adjusted for age, body mass index, smoking status, alcohol drinking status, and physical activity.

## Data Availability

The data presented in this study are available on request from the corresponding author.

## Supplementary Materials

The following supporting information can be downloaded at: https://www.mdpi.com/article/10.3390/nu14122460/s1, Table S1: Factor loadings of the dietary patterns derived from factor analysis.
