# Supplementary material for: Association among Premenstrual Syndrome, Dietary Patterns, and Adherence to Mediterranean Diet
Source: Nutrients. 2022 Jun 14;14(12):2460. doi: 10.3390/nu14122460 (PMC9230049; doi:10.3390/nu14122460)
Supplement: Supplementary file 1 [file nutrients-14-02460-s001.zip › nutrients-1761213-supplementary.pdf]

Supporting information

Supplementary Table S1. Factor loadings of the dietary patterns derived from factor analysis

Variables	Traditional diet pattern	Meat and alcohol pattern	Bread and snack pattern
**Rice**	-0.018	**0.649**	-0.020
**Noodles/dumplings**	0.000	**0.528**	0.401
**Breads/rice cakes**	0.100	0.139	**0.830**
**Soup/stew**	0.571	**0.665**	0.117
**Eggs**	0.452	0.031	0.416
**Red meat**	0.408	**0.666**	0.196
**White meat**	0.066	0.332	0.361
**Fish/seafood**	**0.623**	0.389	0.134
**Vegetables**	**0.656**	0.263	0.250
**Kimchi**	**0.578**	0.190	-0.098
**Seaweed**	**0.694**	-0.120	0.041
**Potatoes**	**0.732**	0.096	0.041
**Fruits**	**0.600**	-0.305	0.361
**Snacks**	0.102	0.161	**0.721**
**Alcoholic beverages**	0.017	0.280	0.181
**Variance explained (%)**	21.7	14.6	13.2
